# Risk factors for cerebral palsy in neonates due to placental abruption

**DOI:** 10.1111/jog.14447

**Published:** 2020-09-03

**Authors:** Kiyotake Ichizuka, Satoshi Toyokawa, Tsuyomu Ikenoue, Shoji Satoh, Junichi Hasegawa, Tomoaki Ikeda, Nanako Tamiya, Akihito Nakai, Keiya Fujimori, Tsugio Maeda, Naohiro Kanayama, Hideaki Masuzaki, Mitsutoshi Iwashita, Hideaki Suzuki, Satoru Takeda

**Affiliations:** ^1^ Department of the Japan Obstetric Compensation System for Cerebral Palsy in Public Interest Incorporated Foundation Japan Council for Quality Health Care Tokyo Japan; ^2^ Department of Obstetrics and Gynecology Showa University Northern Yokohama Hospital Yokohama Japan; ^3^ Department of Public Health, Graduate School of Medicine University of Tokyo Tokyo Japan; ^4^ Department of Obstetrics and Gynecology, Faculty of Medicine Miyazaki University Miyazaki Japan; ^5^ Department of Obstetrics Oita Prefectural Hospital Oita Japan; ^6^ Department of Obstetrics and Gynecology Saint Marianna University School of Medicine Kawasaki Japan; ^7^ Department of Obstetrics and Gynecology Mie University Tsu Japan; ^8^ Department of Health Services Research, Faculty of Medicine Tsukuba University Tsukuba Japan; ^9^ Department of Obstetrics and Gynecology Nippon Medical University Tokyo Japan; ^10^ Department of Obstetrics and Gynecology Fukushima Medical University Fukushima Japan; ^11^ Maeda Obstetrics and Gynecology Clinic Yaizu Japan; ^12^ Department of Obstetrics and Gynecology Hamamatsu University School of Medicine Hamamatsu Japan; ^13^ Department of Obstetrics and Gynecology Nagasaki University Nagasaki Japan; ^14^ Kugayama Hospital Tokyo Japan; ^15^ Department of Obstetrics and Gynecology Juntendo University Tokyo Japan

**Keywords:** alcohol consumption, cerebral palsy, placental abruption, risk factors, smoking

## Abstract

**Aim:**

This study aimed to identify risk factors for the onset of cerebral palsy (CP) in neonates due to placental abruption and investigate their characteristics.

**Methods:**

A retrospective case–control study was conducted using a nationwide registry from Japan. The study population included pregnant women (*n* = 122) who delivered an infant with CP between 2009 and 2015, where placental abruption was identified as the single cause of CP. The control group consisted of pregnant women with placental abruption, who delivered an infant without CP and were managed from 2013 to 2014. They were randomly identified from the prenatal database of the Japan Society of Obstetrics and Gynecology (JSOG‐DB; *n* = 1214). Risk factors were investigated using multivariate analysis.

**Results:**

Alcohol consumption (3.38, 2.01–5.68) (odds ratio, 95% confidence interval), smoking during pregnancy (3.50, 1.32–9.25), number of deliveries (1.28, 1.05–1.56), polyhydramnios (5.60, 1.37–22.6), oral administration of ritodrine hydrochloride (2.09, 1.22–3.57) and hypertensive disorders in pregnancy (2.25, 1.27–4.07) were significant risk factors. In contrast, intravenous administration of oxytocin (odds ratio, 95% confidence interval: 0.22, 0.09–0.58) and magnesium sulfate (0.122, 0.02–0.89) attenuated risk.

**Conclusion:**

Alcohol consumption, smoking during pregnancy, number of deliveries, polyhydramnios, oral administration of ritodrine hydrochloride and hypertensive disorders in pregnancy were identified as risk factors for CP following placental abruption. Regarding alcohol consumption and smoking during pregnancy, the results suggest the importance of educational activities targeting pregnant women to increase their awareness of placental abruption.

## Introduction

Placental abruption is an uncommon condition where the placenta detaches from the inner wall of the uterus before birth. The reported frequency of placental abruption is approximately 5.9 in 1000 deliveries in singleton pregnancies and 12.2 in 1000 in twin pregnancies. The rate of perinatal mortality in pregnancies complicated by placental abruption is more than 10 times higher than the overall perinatal mortality rate.^1^, ^2^, ^3^ In Japan, it has been reported that the adjusted relative risk of cerebral palsy (CP) due to placental abruption is 20.891 (95% confidence interval [CI]: 11.817–36.934).^4^ Hypertensive disorders in pregnancy, a history of placental abruption, intra‐amniotic infection, preterm labor, preterm rupture of membranes, trauma, smoking and alcohol consumption are known risk factors for placental abruption.^1^, ^2^, ^5^ Yamada *et al*.^6^ reported that placental abruption was the causative factor in 28 (26%) of 107 infants with CP, making it the single leading causative factor. However, there have been no studies on prepartum risk factors in cases of CP due to placental abruption. If other risk factors for placental abruption can be addressed, the actual incidence of CP due to placental abruption may decrease. This study aimed to identify risk factors for the onset of CP due to placental abruption and investigate their characteristics.

## Methods

A retrospective case–control study was conducted using the perinatal database of the Japan Society of Obstetrics and Gynecology (JSOG‐DB), which is the largest registry in Japan. Obstetric clinical characteristics and obstetric risk factors were compared between the CP cases (*n* = 122) and control cases (*n* = 1214).

The CP cases comprised infants with CP for whom compensation was approved in a review by the operating organization of the Japan Obstetric Compensation System for Cerebral Palsy (JOCSC). The JOCSC provides prompt no‐fault compensation for children diagnosed with severe CP caused by trauma during labor and delivery, as well as for their respective families. Compensation cases are reviewed by a committee consisting of obstetricians, pediatricians, midwives and lawyers, according to the rules of the Operating Organization of the JOCSC. After being deemed as eligible to receive compensation by this review committee, the causes of CP are analyzed individually by the Causal Analysis Committee, which consists of obstetricians, pediatricians, midwives and lawyers.

Cases involving pregnant women who delivered an infant with CP, in which placental abruption was identified as the single cause of CP, were eligible for inclusion in the present study (*n* = 122). All infants were born between January 2009 and December 2015 and had a birth weight of ≥2000 g, gestational age of ≥33 weeks and severe disability due to CP (unassociated with congenital causes or factors that occurred during the neonatal period or later), with a disability certified as first‐ or second‐degree severity according to the definitions in the Act for the Welfare of Persons with Physical Disabilities (https://www.dinf.ne.jp/doc/english/resource/z00009/z0000901.html).

**Figure 1:**
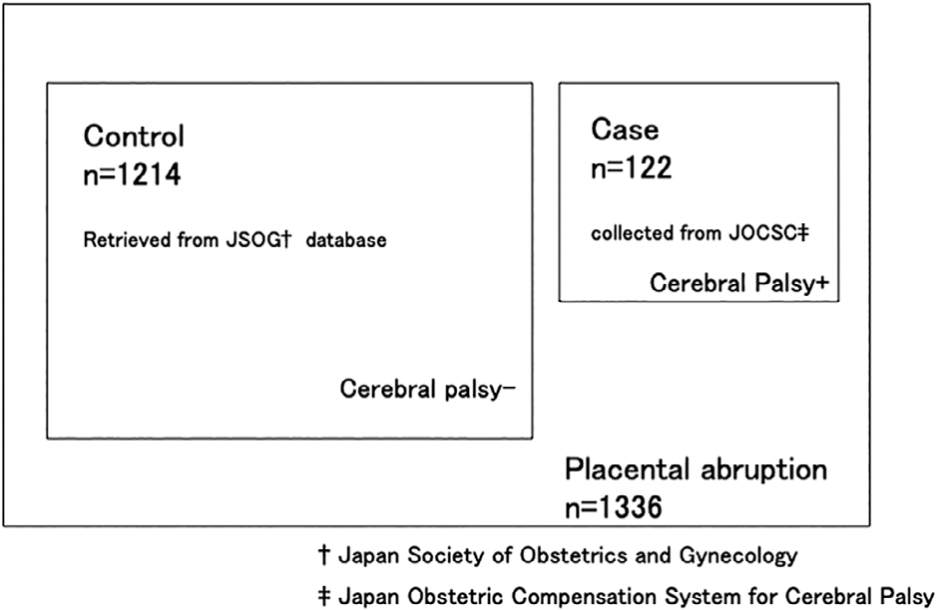
Study participants. †Japan Society of Obstetrics and Gynecology. ‡Japan Obstetric Compensation System for Cerebral Palsy.

The control participants' data were extracted from the perinatal database of the JSOG‐DB, the largest registry in Japan established in 1974. The JSOG‐DB accumulates data annually from 192 secondary and tertiary care centers of the Perinatal Research Network in Japan, recording approximately 24.9% of total births and approximately 51.6% of perinatal deaths. It collects data on each pregnant woman through an offline clinical database system using a common format, which are then stored with strict quality control of the information.^7^ It is available to clinical researchers in Japan and has been used in similar studies.^4^, ^6^, ^8^, ^9^ In this study, the control group, included pregnant women with placental abruption who delivered an infant without CP between 2013 and 2014. Control participants (*n* = 1214) were randomly retrieved from the JSOG‐DB. The data of all participants (*n* = 1336) are shown in Figure 1.

The statistical power asymptotically increases by the number of matching controls; 10 controls per each CP case are sufficient to obtain converged statistical power. We avoided using all control candidates from the database because erroneous data inputted by mistake might be included in the analysis dataset. Fortunately, the results using a 1:10 matching control ratio and the entire control cohort were identical.

The explanatory variables in this study were age, height, prepregnancy body weight, bodyweight increase during pregnancy, alcohol consumption during pregnancy, smoking before and during pregnancy, multiparity, diabetes mellitus, thyroid disease, uterine fibroids, assisted reproductive technology (ART), oligohydramnios, polyhydramnios, oral administration of ritodrine hydrochloride, intravenous administration of ritodrine hydrochloride, intravenous administration of magnesium sulfate, intravenous administration of oxytocin, oral administration of aspirin, intramuscular injection of betamethasone, preterm rupture of membranes, intrauterine infection, preterm labor, hypertensive disorders in pregnancy and gestational age.

### Statistical analysis

Relationships among clinical variables were evaluated using univariate and multivariate logistic regression analyses with CP as the independent variable. Results are expressed as odds ratios (ORs) and 95% CIs. Univariate and multivariate analyses were performed using explanatory variables to calculate the crude odds ratios (cORs) and adjusted odds ratios (aORs). The significant explanatory variables that were identified in univariate analysis and variables considered to be clinically important, such as maternal age, height, bodyweight before pregnancy, body weight increase during pregnancy and birth weight, were entered into a multivariate model. Two‐sided *P* values <0.05 were considered statistically significant. All analyses were conducted using stata version 13.0 (STATA Corporation).

### Definitions

#### *Cerebral palsy*

Cerebral palsy was defined as a disturbance of the motor function or posture of infants that is permanent or variable. The disorder is based on a nonprogressive cerebral lesion that may develop at any time between conception and the neonatal period (within 4 weeks after birth). However, this definition excludes motor retardation, which is either transient or normalizes in the future.

#### *Placental abruption*

In this study, placental abruption was diagnosed according to the judgment of the attending physician based on the clinical course, such as abdominal pain with bleeding, abnormal fetal heart pattern on cardiotocography, the existence of retroplacental hematoma on ultrasonography and other clinical findings.

### Ethics

The study protocol was approved by the institutional review board of the JOCSC (approval number: 26–1). Written informed consent was not obtained from the patients, as this was a retrospective study. However, patients were provided with a supplemental file that contained the announcement of the implementation of a ‘case–control study for cerebral palsy and prevention of its recurrence’. Although the analysis was retrospective, the anonymized data of the CP patients, retrieved from the JOCSC‐DB, and those of the control cohort, retrieved from the JSOG‐DB, had been collected in a normal clinical setting, ensuring that patient confidentiality was protected. All the patient data were anonymized and de‐identified before the analysis. No personal information was necessary for the present study.

This study does not violate the policies and procedures of the journal and conforms to the provisions of the Declaration of Helsinki (as revised in Tokyo, 2004).

## Results

Data for 122 patients with CP were retrieved from the JOCSC‐DB, and data for 1214 controls were randomly retrieved from the JSOG‐DB. The demographic variables are shown in Table 1.

**Table 1:** Demographics of the cerebral palsy and control groups

	Control				Case			
	*n* = 1214				*n* = 122			
	Mean	SD	Min.	Max.	Mean	SD	Min.	Max.
Age	32.5	5.18	16	48	32.1	5.1	19	44
Height	158.1	5.62	136.2	177	158.1	5.82	145	176
Body weight before pregnancy	53.1	9	35	102	53.8	9.3	37	104
Body weight before delivery	62	8.8	40.1	99.1	63.2	9.3	45	107.5
BMI	21.2	3.4	14.8	40	21.5	3.5	16.2	41.6
Body weight gaining	8.87	4.42	−14	35	9.3	3.87	−2.3	18.3
Birth weight	2682.2	401.3	2002	4192	2684.3	396.1	2030	3736
Apgar score 1 min	6.7	2.3	0	10	0.95	1.1	0	6
Apgar score 5 min	8.2	1.6	0	10	2.2	2.2	0	8
Umbilical artery blood pH	7.221	0.14	6.512	7.55	6.719	0.15	6.541	7.165

BMI, body mass index.

The explanatory variables that may be associated with risk factors for placental abruption in the CP and control groups are shown in Table 2. A univariate analysis of the explanatory variables identified alcohol consumption (3.59, 2.25–5.72, <0.01) (cORs, 95% CIs, *P*‐value), smoking before pregnancy (1.77, 1.09–2.88, 0.022), smoking during pregnancy (3.17, 1.66–6.07, <0.01), the number of births (the risk increasing with each additional delivery; 1.37, 1.16–1.61, <0.001), multiparity (1.84, 1.25–2.71, 0.002), oral administration of ritodrine hydrochloride (2.71, 1.84–4.00, <0.001), preterm labor (1.83, 1.24–2.69, 0.002) and hypertensive disorders in pregnancy (1.83, 1.20–3.03, 0.0019) as significant risk factors for CP. Among these, alcohol consumption during pregnancy showed the strongest association with CP. In contrast, the intravenous administration of magnesium sulfate (0.122, 0.02–0.89, 0.004) and the intravenous administration of oxytocin (0.23, 0.09–0.56, 0.001) significantly attenuated the risk of developing CP (Table 3).

**Table 2:** Assessment of obstetric factors in the cerebral palsy and control groups

	Case		Control	
	*n* = 122		*n* = 1214	
Alcohol consumption during pregnancy	29	23.8%	97	8.0%
Smoking before pregnancy	23	18.9%	141	11.6%
Smoking during pregnancy	13	10.7%	44	3.6%
Diabetes mellitus	1	0.8%	67	5.5%
Gestational diabetes mellitus	1	0.8%	60	4.9%
Thyroid disease	1	0.8%	32	2.6%
Uterine fibroid	6	4.9%	63	5.2%
Number of birth 1	43	35.2%	408	33.6%
Number of births 2	26	21.3%	145	11.9%
Number of births 3	5	4.1%	36	4.0%
Number of births 4	5	4.1%	13	1.1%
Number of births 5	0	0.0%	3	0.2%
Number of births 6	0	0.0%	1	0.1%
Number of births 7	0	0.0%	1	0.1%
Multiparity	79	64.8%	607	50.0%
Assisted reproductive technology (ART)	1	0.8%	66	5.4%
Polyhydramnios	3	2.5%	9	0.7%
Oligohydramnios	3	2.5%	27	2.2%
Oral administration of ritodrine hydrochloride	49	40.2%	241	19.9%
Intravenous administration of ritodrine hydrochloride	13	10.7%	195	16.1%
Intravenous administration of magnesium sulfate	1	0.8%	77	6.3%
Oral administration of aspirin	1	0.8%	19	1.6%
Intramuscular injection of betamethasone	0	0.0%	16	1.3%
Preterm rupture of membranes	17	13.9%	147	12.1%
Intravenous administration of oxytocin	5	4.1%	194	16.0%
Intrauterine infection	12	9.8%	105	8.6%
Preterm labor	47	38.5%	310	25.5%
Hypertensive disorder in pregnancy	21	17.2%	124	10.2%
Gestational age (week)				
33	4	3.3%	30	2.5%
34	7	5.7%	83	6.8%
35	16	13.1%	135	11.1%
36	15	12.3%	171	14.1%
37	28	23.0%	206	17.0%
38	26	21.3%	235	19.4%
39	16	13.1%	192	15.8%
40	7	5.7%	125	10.3%
41	3	2.5%	37	3.0%
42	0	0.0%	1	0.1%

**Table 3:** Univariate analysis of obstetric factors identified in the cerebral palsy and control groups

	Univariate analysis		
	cORs	95%CIs	*P*‐value
Alcohol consumption during pregnancy	3.591	2.254–5.720	<0.001
Smoking before pregnancy	1.768	1.087–2.876	0.022
Smoking during pregnancy	3.171	1.657–6.069	<0.001
Diabetes mellitus	0.141	0.019–1.028	0.053
Gestational diabetes mellitus	0.159	0.022–1.157	0.069
Thyroid disease	0.305	0.041–2.254	0.245
Uterine fibroid	0.945	0.400–2.231	0.897
Number of births (increased every once)	1.365	1.155–1.613	<0.001
Multiparity	1.837	1.246–2.708	0.002
Assisted reproductive technology (ART)	0.144	0.020–1.045	0.055
Polyhydramnios	3.375	0.902–12.637	0.071
Oligohydramnios	1.108	0.331–3.708	0.867
Oral administration of ritodrine hydrochloride	2.710	1.838–3.996	<0.001
Intravenous administration of ritodrine hydrochloride	0.623	0.347–1.130	0.119
Intravenous administration of oxytocin	0.225	0.091–0.557	0.001
Intravenous administration of magnesium sulfate	0.122	0.017–0.885	0.004
Oral administration of aspirin	0.520	0.069–1.917	0.525
Intramuscular injection of betamethasone	1.000		
Preterm rupture of membranes	1.175	0.684–2.018	0.558
Intrauterine infection	1.152	0.615–2.160	0.659
Preterm labor	1.827	1.241–2.690	0.002
Hypertensive disorder in pregnancy	1.828	1.203–3.030	0.019
Gestational age (increased every week)	0.943	0.857–1.037	0.228

cOR, crude odds ratio; CIs, confidence intervals.

**Figure 2:**
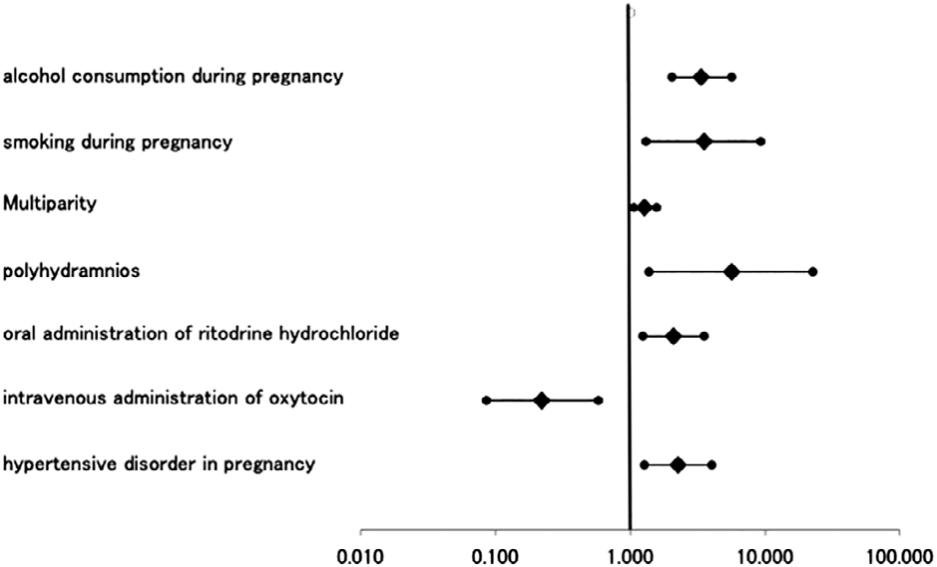
Forest plots of multivariate analysis. All variables are statistically significant. 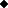 represents aORs. Lines between 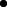 to 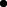 represents 95% CIs.

In the multivariate analysis, alcohol consumption (3.38, 2.01–5.68, <0.001) (aORs, 95% CIs, *P*‐value), smoking during pregnancy (3.50, 1.32–9.25, 0.012), multiparity (1.28, 1.05–1.56, 0.011), polyhydramnios (5.60, 1.37–22.6, 0.016), oral administration of ritodrine hydrochloride (2.09, 1.22–3.57, 0.007) and hypertensive disorders in pregnancy (2.25, 1.27–4.07, 0.006) were identified as significant independent risk factors. Intravenous administration of oxytocin significantly attenuated the risk of developing CP (0.22, 0.09–0.58. 0.002) (OR, 95% CI) (Table 4, Figure 2).

**Table 4:** Multivariate analysis of all variables

	Multivariate analysis		
	aORs	95% CIs	*P*‐value
Age	0.972	0.933–1.012	0.172
Height	0.984	0.947–1.023	0.426
Body weight before pregnancy	1.011	0.986–1.036	0.396
Body weight increase during pregnancy	1.033	0.983–1.086	0.199
Birth weight	1.003	0.999–1.006	0.125
Birth weight SD	0.444	0.140–1.404	0.167
Alcohol consumption during pregnancy	3.383	2.013–5.685	<0.001
Smoking before pregnancy	1.481	0.350–1.542	0.415
Smoking during pregnancy	3.495	1.321–9.250	0.012
Multiparity	1.284	1.059–1.558	0.011
Polyhydramnios	5.604	1.374–22.865	0.016
Oligohydramnios	1.188	0.320–4.413	0.797
Oral administration of ritodrine hydrochloride	2.093	1.226–3.573	0.007
Intravenous administration of ritodrine hydrochloride	0.509	0.256–1.012	0.054
Intravenous administration of magnesium sulfate	0.136	0.018–1.029	0.053
Intravenous administration of oxytocin	0.223	0.086–0.580	0.002
Intrauterine infection	1.063	0.535–2.113	0.862
Preterm labor	1.562	0.879–2.777	0.128
Hypertensive disorder in pregnancy	2.252	1.265–4.010	0.006
Gestational age	0.657	0.382–1.130	0.224

aOR, adjusted odds ratio; CI, confidence intervals.

## Discussion

Cerebral palsy, especially that originating in the intrapartum period, not only impacts the patient and parents but is also a significant medicolegal matter.^10^ In the last two decades, medicolegal claims arising from perinatal brain injury have continued growing.^11^ In Japan, obstetrics‐related lawsuits filed between 2004 and 2008 accounted for approximately 10–15% of the overall total across all specialties. However, since the institution of this system, obstetrics‐related lawsuits have started to fall to approximately 7% of the total.^12^ Nevertheless, CP remains the subject of lawsuits, and improving prevention is an important obstetrical issue. For that reason, it is necessary to investigate its cause and risk factors. Against this background, various studies have investigated the cause of CP and its risk factors.^10^, ^13^ Previous research has identified placenta previa as one of the leading causes of CP.^13^

In this study, we found that the umbilical cord blood pH was significantly lower after placenta abruption in the CP group than in the non‐CP group. Moreover, we elucidated the risk factors for CP occurring during pregnancy following a placental abruption.

A study that analyzed the umbilical cord blood pH revealed acidemia (pH ≤7.0) in 114 of 168 (69.2%), neonates, with severe CP and in 42 of 42 (100%) fetuses with CP in cases involving placental abruption.^14^ According to a report by the JOCSC, more than half of all CP cases showed umbilical cord blood pH of less than 7.0,^15^ indicating that fetal acidemia is closely related to CP. Our cohort was also derived from the JOCSC database, which only includes CP due to causes related to pregnancy and delivery, excluding congenital malformations or postnatal causes. Another report found that a pH <7.0 was an essential criterion in defining the cause as intrapartum.^16^, ^17^ Matsuda *et al*.^18^ reported that fetal bradycardia is the most important risk factor for CP among patients with a fetal heart‐tracing pattern. Conversely, there are no reports on risk factors related to the onset of CP in patients with placental abruption. Alcohol drinking, smoking during pregnancy, multiparity, polyhydramnios and hypertensive disorders in pregnancy have previously been reported as risk factors for placental abruption,^1^, ^2^, ^3^ and are also risk factors for the development of CP after placental abruption.

In the present study's multivariate analysis, alcohol consumption was a risk factor for CP after placental abruption. However, alcohol consumption, especially heavy consumption, may be a cause of neurodevelopmental abnormalities, including CP.^19^ Smoking was examined separately, before and during pregnancy. The univariate analysis identified smoking before and during pregnancy as risk factors; however, in the multivariate analysis, only smoking during pregnancy remained significant. This suggests that smoking before pregnancy was tolerable and that smoking cessation during pregnancy could reduce the risk of CP due to placental abruption. This information would be useful for the education of pregnant women. The oral administration of ritodrine hydrochloride was identified as a prepartum risk factor. The reason for this may be that medication is administered outside the hospital setting, where fetal well‐being cannot be frequently confirmed, and symptoms may be masked by the medication. Consequently, delayed detection of placental abruption rather than the direct effect of a drug such as ritodrine hydrochloride may be associated with the increased risk of developing CP. Furthermore, in this study, we could not detect a pharmacological effect of ritodrine hydrochloride against the occurrence of placental abruption. Thus, when patients take ritodrine hydrochloride for the management of preterm labor in an outpatient setting, it is important to instruct them on how to respond to changes such as decreased fetal movement, sudden abdominal pain, continuous pain or excessive bleeding, before they start treatment.

Preterm labor showed an cORs of 1.83 in the univariate analysis (*P* = 0.002) but did not remain significant in the multivariate analysis. However, making an accurate differential diagnosis between preterm labor and placental abruption is important because, although their clinical symptoms are very similar, their prognoses are very different. In particular, special attention must be paid to pregnant women on oral ritodrine hydrochloride for preterm labor, who may have already developed placental abruption.

The intravenous administration of oxytocin was identified as a factor that significantly attenuated the risk of developing CP. However, this could be because continuous fetal heart rate tracing led to the early detection of fetal pathological conditions such as a nonreassuring status. The intravenous administration of magnesium sulfate was also identified as a significant factor in the univariate analysis but was not found to significantly attenuate the risk of developing CP in the multivariate analysis. In case of preterm delivery (≤33 + 6 weeks), administration of magnesium sulfate has been reported as a fetal neuroprotective, and its antenatal use is recommended for neuroprotection of the preterm infant.^20^ In this study, intravenous magnesium sulfate was not a significant risk factor, because almost all cases had a gestation period of more than 34 weeks.

Smoking before pregnancy was not a risk factor for placental abruption; however, smoking during pregnancy was a risk factor. This suggests that smoking during pregnancy can reduce the risk of premature detachment, and it is important to educate women who smoke about this risk.

The present study had some limitations. Since the case and control groups were collected from the different 4‐year periods, this may lead to bias related to different practices during different periods. However, during these different periods, critical practices regarding placental abruption were not different. Therefore, collecting data at different periods might have minimal impact. The diagnostic criteria for placental abruption are not uniform. The diagnosis of placental abruption may be biased because it was solely made by the attending physician. It is possible that there was a facility bias because the control cases were extracted from the JSOG‐DB. The JSOG‐DB is dominated by secondary and tertiary care facilities, while the CP group was dominated by patients from primary care facilities. Thus, the diagnosis of placental abruption may be different between groups with and without CP. As the study did not include infants weighing under 2000 g or cases of fetal death, the risk factors may differ in cases involving more immature fetuses and those with a poor prognosis. Cerebral palsy has a strong link with gestational age. In this study, cases and controls included not only preterm but also term pregnancies. On the other hand, both groups were not different in gestational age distribution. However, to reduce the bias of the gestational age, cases with CP should be matched to the controls with abruption but without CP by gestational age. This issue is one of the significant limitations of this study.

In conclusion, risk factors for the development of CP after placental abruption were identified. Risk factors such as alcohol consumption, smoking during pregnancy and oral administration of ritodrine hydrochloride were included among those that could be ameliorated through educating pregnant women. Medical intervention for preterm labor, especially in the outpatient setting, should be performed appropriately. This includes confirming fetal well‐being. The findings suggest the importance of educational activities for pregnant women to increase their awareness of placental abruption and its possible effects.

## Disclosure

None declared.

## Author contributions

Since the content of this paper was discussed and compiled by an organization of 15 people, the number of authors, including co‐authors, will be 15.
